# Inappropriate treatment of pulmonary aspergillosis caused by *Aspergillus flavus* in susceptible pediatric patients: a case series

**DOI:** 10.1186/s13256-024-04599-9

**Published:** 2024-07-02

**Authors:** Neginsadat Hosseinikargar, Hossein Zarrinfar, Seyed Javad Seyedi, Seyedeh Sabereh Mojtahedi

**Affiliations:** 1 Student Research Committee, Mashhad University of Medical Sciences, Mashhad, Iran; 2 Allergy Research Center, Mashhad University of Medical Sciences, Mashhad, Iran; 3 Sinus and Surgical Endoscopic Research Center, Mashhad University of Medical Sciences, Mashhad, Iran; 4 Department of Pediatrics, Faculty of Medicine, Mashhad University of Medical Sciences, Mashhad, Iran; 5 Department of Parasitology and Mycology, School of Medicine, Mashhad University of Medical Sciences, Mashhad, Iran; 6 Department of Laboratory Sciences, School of Paramedical Sciences, Mashhad University of Medical Sciences, Mashhad, Iran

**Keywords:** Pulmonary aspergillosis, Pediatric patients, Antifungal, *Aspergillus*

## Abstract

**Background:**

Pulmonary aspergillosis is a prevalent opportunistic fungal infection that can lead to mortality in pediatric patients with underlying immunosuppression. Appropriate and timely treatment of pulmonary aspergillosis can play a crucial role in reducing mortality among children admitted with suspected infections.

**Case presentation:**

The present study reports three cases of inappropriate treatment of pulmonary aspergillosis caused by *Aspergillus flavus* in two Iranian pediatric patients under investigation and one Afghan patient. Unfortunately, two of them died. The cases involved patients aged 9, 1.5, and 3 years. They had been diagnosed with pulmonary disorders, presenting nonspecific clinical signs and radiographic images suggestive of pneumonia. The identification of *A*. *flavus* was confirmed through DNA sequencing of the calmodulin (*CaM*) region.

**Conclusion:**

*A*. *flavus* was the most prevalent cause of pulmonary aspergillosis in pediatric patients. Early diagnosis and accurate antifungal treatment of pulmonary aspergillosis could be crucial in reducing the mortality rate and also have significant potential for preventing other complications among children. Moreover, antifungal prophylaxis seems to be essential for enhancing survival in these patients.

## Introduction

*Aspergillus* species are commonly found in the environment, and inhaling their conidia can lead to invasive diseases in immunocompromised individuals, especially in pediatric patients [1]. *Aspergillus flavus* is a major contributor to life-threatening invasive aspergillosis (IA) in the Middle East, primarily affecting immunocompromised patients [2, 3]. Children and adults have similar disease presentations, distributions, patterns, and susceptibility to pulmonary aspergillosis (PA). However, there are variations in the pharmacology of antifungal medications, the epidemiology of underlying diseases, and the use of improved diagnostic methods [4]. Early diagnosis and treatment will enhance outcomes, particularly in neonates and pediatric patients [5]. Diagnosing pulmonary aspergillosis is challenging because recovering *Aspergillus* from respiratory specimens cannot differentiate between colonization and invasion [6, 7]. In some forms of PA, such as chronic fibrosing PA (CFPA), bronchiectasis, and other associated changes in the lungs will occur. In patients with pulmonary issues, it is strongly recommended to perform chest computed tomography and bronchoscopy with bronchoalveolar lavage (BAL) [8]. BAL is also beneficial for evaluating pediatric lung diseases and can be essential for detecting respiratory infections, particularly PA [6, 8]. It is strongly recommended that all clinically relevant *Aspergillus* isolates undergo pathogen identification at the species complex level [9]. Appropriate and timely treatment of PA can play an important role in reducing mortality among vulnerable patients [10]. This study describes three cases of inappropriate treatment of pulmonary aspergillosis caused by *A*. *flavus* in pediatric patients from Iran in the Middle East.

## Case 1

**Fig. 1:**
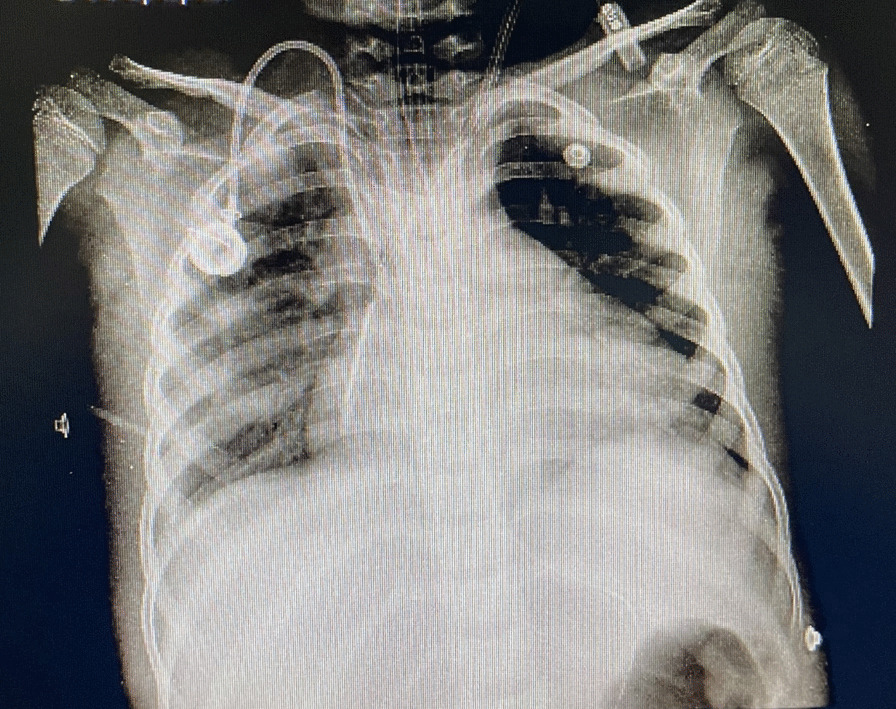
A chest radiograph showing an opaque patch in the peripheral right hemithorax of the lungs

In April 2021, a 9-year-old Afghan boy was admitted to Sheikh Hospital in Mashhad. He presented with a fever, dyspnea, nonproductive cough, and respiratory distress. Additionally, he had previously received chemotherapy for Hodgkin’s lymphoma and tested negative for coronavirus disease 2019 (COVID-19) upon admission. Although he was treated with vancomycin and meropenem for antibiotic prophylaxis, he had not received any antifungal prophylaxis. Additionally, the patient received a blood transfusion. The radiography scans revealed the following outcomes: in the lung radiography scan (Fig. 1), an opacity patch was observed in the peripheral right hemothorax of the lungs. During his hospitalization, he also underwent a total gastrectomy and an intestinal biopsy. His hematological findings showed that his white blood cell (WBC) count was 0.5 × 10^3^/μl, red blood cell (RBC) count was 2.56 × 10^6^/μl, hemoglobin (Hb) was 6.9 g/dl, hematocrit (HCT) was 21.4%, platelet (PLT) count was 52 × 10^3^/μl, mean corpuscular volume (MCV) was 83.59 fl, mean corpuscular hemoglobin (MCH) was 26.95 pg, and mean corpuscular hemoglobin concentration (MCHC) was 32.24 g/dl. Moreover, other laboratory findings showed that the uric acid level was 4.2 mg/dl, and levels of calcium, phosphorus, urea, creatinine, sodium, and potassium were within the normal range. After the bronchoscopy, the secretions were sent to the medical mycology lab for additional diagnostic testing. The *Pneumocystis jirovecii* test result was negative for this patient. The clinical specimen was examined microscopically using 15% potassium hydroxide (KOH), and several hyaline septate hyphae were observed. In addition, the clinical specimen was also cultured on Sabouraud dextrose agar (SDA) and then incubated for 4–6 days at 35 °C. The colonies exhibited a yellowish-green appearance surrounded by a white circle that was eventually covered by conidia, as revealed by microscopic examination, indicating an *Aspergillus* species. The genomic DNA of *Aspergillus* was extracted, and polymerase chain reaction (PCR) and Sanger sequencing of the calmodulin (*CaM*) region were performed as described previously [11]. The closest match to the isolate in the *CaM* BLAST in GenBank was *A*. *flavus*. The genome sequence of the isolate has been deposited in GenBank with the accession number OQ538375. He had received imipenem, cefuroxime, and acyclovir for pulmonary pneumonia. Regrettably, owing to a delayed diagnosis of PA and a lack of prompt treatment with antifungal medication, the patient passed away after 22 days of hospitalization.

## Case 2

**Fig. 2:**
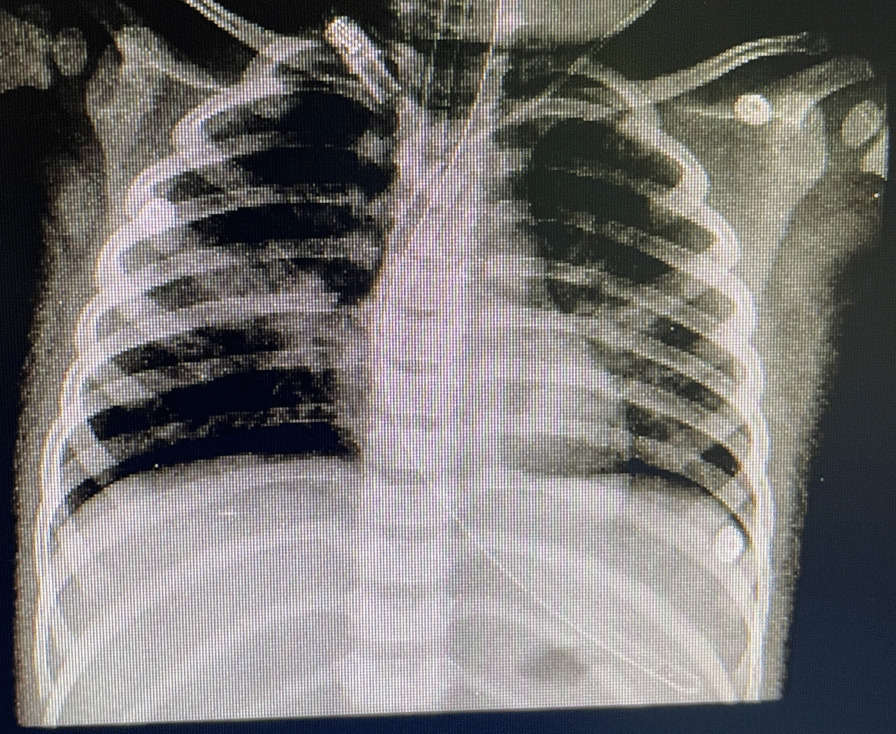
A chest radiograph showing consolidation in the middle zone of the right lung during a lung radiography scan

In December 2021, a 1.5-year-old Iranian girl from Chenaran, located in Khorasan Razavi, was admitted to Sheikh Hospital in Mashhad. She had drowned in a pool for 10 minutes. Thereafter, she was resuscitated for 25 minutes, but her blood pressure and blood sugar levels were elevated. Additionally, she also received insulin. After her blood sugar levels returned to normal, she was administered dopamine. The patient was catheterized and intubated. Upon arrival at the hospital, she was unconscious and using an artificial manual breathing unit (Ambu) bag. She also experienced diarrhea, vomiting, and fever, and blood secretions were observed from the anus. However, she only received clindamycin for prophylaxis. Her hematological findings showed a WBC count of 2.7 × 10^3^/μl, RBC count of 4.47 × 10^6^/μl, Hb of 12.8 g/dl, HCT of 39.1%, PLT count of 152 × 10^3^/μl, MCV of 87.47 fl, MCH of 28.64 pg, and MCHC of 32.74 g/dl. The patient’s blood group was B+, and she received a transfusion. Her COVID-19 test result was negative. The radiography scan revealed consolidation in the middle zone of the right lung (Fig. 2). After a bronchoscopy, the BAL specimen was sent to the medical mycology laboratory for further analysis. The *P*. *jirovecii* test result was negative for this patient. The clinical specimen was examined microscopically using 15% KOH, which revealed septate hyphae similar to those in case 1. Another portion of the clinical specimen was also cultured on a SDA plate and then incubated at 35 °C for 4–6 days. The macroscopic and microscopic examination of the colonies revealed an *Aspergillus* species. The genomic DNA of *Aspergillus* was extracted, and PCR and Sanger sequencing of the *CaM* region were performed as previously described [11]. The closest match to the isolate in the *CaM* BLAST in GenBank was *A*. *flavus*. The genome sequence of the isolate has been deposited in GenBank with the accession number OQ538376. Furthermore, she was treated with ceftriaxone antibiotics. Regrettably, as a result of the delayed diagnosis and the severity of the PA infection, coupled with the absence of prompt treatment with antifungal medication, the patient passed away 3 days after being admitted to the hospital.

## Case 3

**Fig. 3:**
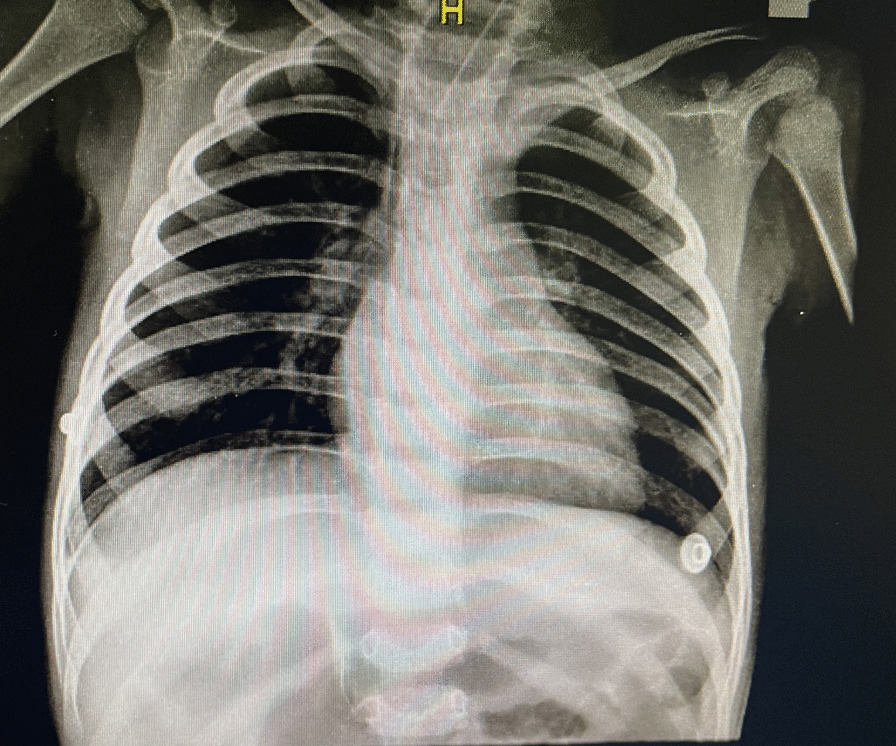
A chest radiograph showing lung involvement in the right peripheral area

A 3-year-old Iranian girl was admitted to Sheikh Hospital in Mashhad in June 2021. She presented with fever, a nonproductive cough, convulsions, diarrhea, vomiting, lethargy, weakness, and signs of cerebral palsy (CP). She was diagnosed with pneumonia. However, the physician prescribed vancomycin and metronidazole as prophylaxis. The patient underwent catheterization and received a blood transfusion. Her COVID-19 test result was negative. The lung radiography scan revealed lung involvement in the right peripheral area (Fig. 3). Her hematological findings showed a WBC count of 9.8 × 10^3^/μl, RBC count of 3.64 × 10^6^/μl, Hb of 10 g/dl, HCT of 31%, PLT count of 331 × 10^3^/μl, MCV of 85 fl, MCH of 27 pg, MCHC of 32 g/dl, urea of 36 mg/dL, and high C-reactive protein (CRP) levels. Additionally, the levels of calcium, uric acid, creatinine, sodium, and potassium were within the normal range. After bronchoscopy, the BAL specimen was sent to the medical mycology laboratory for examination of fungal infections. The result of the *P*. *jirovecii* test, using real-time PCR, was positive for this patient. The septate hyphae were observed in a direct examination of the BAL specimen using 15% KOH. Additionally, another portion of the BAL specimen was cultured on a SDA plate and then incubated at 35 °C for 4–6 days. *Aspergillus* species were identified using PCR and sequencing, as previously described [11]. The PCR and Sanger sequencing of the *CaM* region of the isolate revealed that the closest match in the *CaM* BLAST in GenBank was *A*. *flavus* species. The genome sequence has been deposited in GenBank with the accession number OQ538374. The patient was prescribed seizure medication and amikacin. Despite only experiencing partial recovery and insisting on being discharged, the patient left the hospital without receiving antifungal drugs. Unfortunately, we were unable to follow up with this patient regarding the fungal infection.

## Discussion

*Aspergillus* infections are a significant cause of morbidity and mortality, particularly among the growing population of immunocompromised patients [12]. Corticosteroids and prolonged neutropenia are known risk factors for this complication. Patients with PA usually present with fever, pleuritic chest pain, and hemoptysis [13]. Pneumothorax due to PA in children is extremely rare. However, it can be a devastating complication in children with hematological disorders [12]. Diagnosing invasive pulmonary aspergillosis (IPA) definitively remains challenging owing to its wide range of clinical features and the absence of approved laboratory methods. The type of clinical specimen, the sensitivity, and specificity of laboratory methods, as well as the availability of these techniques in medical centers, can significantly impact the diagnosis of this disease. Histopathological characteristics are considered the gold standard for diagnosing IPA. However, obtaining a tissue biopsy is often not feasible owing to the fragile condition of the patient, particularly in pediatric cases where invasive procedures may pose a risk. Therefore, the BAL fluid appears to be a relatively safe and useful specimen in high-risk patients suspected of having PA [6]. In contrast, traditional methods have much lower sensitivity compared with molecular and serological methods and cannot definitively diagnose IPA on their own. Yeoh *et al*. conducted a review study that demonstrated the inherent challenges in the timely diagnosis of IPA. They suggested that a combination of computed tomography (CT) imaging and microbiological testing can facilitate this process [14]. In the current study, CT scans were not performed. Instead, the initial diagnosis of these patients relied on chest X-rays and microbiological procedures. In CT and plain radiograph findings, most studies describe nodular opacities as most frequent, followed by wedge-shaped/lobar consolidations. Our cases showed consolidation and opacity patches in the chest X-ray without any wedge shape. However, radiographic images cannot specifically distinguish infections caused by *Aspergillus* from other microbial infections. Yeoh *et al*. also demonstrated that respiratory sampling through either BAL or lung biopsy is recommended, but it is not always feasible in pediatric patients.

Similarly, in our study, the BAL specimen could help identify fungal agents. However, it is important to distinguish cases of colonization from actual invasion of host tissues by fungal agents. Shah *et al*. reported three children who developed pneumothorax as a presenting feature of PA during induction chemotherapy for leukemia [1]. The diagnosis of PA was based on clinical manifestations, radiology findings, and a serum galactomannan test in two cases, and in one case, a specimen obtained by needle aspiration. In this report, we describe cases of pneumothorax in three children. One case had undergone chemotherapy, and the other had a coinfection with *P*. *jirovecii*. Unfortunately, galactomannan test on BAL or serum was not conducted in our cases owing to financial constraints. Crassard *et al*. conducted a 15-year review study in a pediatric hematology department and reported that 22 patients presented with lung involvement related to IPA [15]. The positive culture revealed the presence of various *Aspergillus* species, including *A*. *fumigatus* (18 cases), *A*. *nidulans* (3 cases), *A*. *flavus* (1 case), and *A*. *terreus* (1 case). Two species, *A*. *fumigatus* and *A*. *nidulans*, were isolated in a BAL culture from only one patient. Mark de mol *et al*. suggested that the galactomannan (GM) assay on BAL specimens is a valuable diagnostic tool for detecting IPA in children, with high sensitivity and specificity for the BAL GM index [7]. Several diagnostic studies have shown that the detection of BAL GM has better test performance than serum [16, 17]. They found that 41 children suffered from IPA, diagnosed based on a GM test. During the direct examination, two specimens showed septate hyphae, and the culture results of seven specimens were positive for *Aspergillus* spp. The distribution of positive cultures was as follows: four *A*. *fumigatus*, two *A*. *flavus*, and one *Aspergillus* spp. Regrettably, in the current study, we were unable to conduct the GM test owing to several limitations. Children with IPA typically exhibit nonspecific radiographic findings, in contrast to the cavitary lesions frequently observed in adults [12]. We reported nonspecific radiographic findings, such as patches of opacity and consolidation. In a retrospective multicenter analysis of pediatric IPA, the most common diagnostic radiologic finding was nodules [8]. However, the German acute lymphocytic leukemia (ALL)-Berlin-Frankfurt-Muenster (BFM) study group reported that fungal infections accounted for one-fifth of fatal infections in pediatric patients with ALL [18]. *Aspergillus* was implicated in two-thirds of the cases of invasive fungal infections [18]. Despite the devastating complications and high mortality associated with IA, there is still no consensus on a prophylactic agent or treatment of choice for pediatric patients [19]. In our reports, regrettably, owing to the late diagnosis of PA, these patients did not receive appropriate and timely treatment. Timely diagnosis is crucial in pediatrics because of the potential severity and complications associated with PA. However, the challenges are associated with the nonspecific nature of symptoms and lower yields from microbiological procedures, which can lead to a high mortality rate among pediatric patients. This is because *Aspergillus*, a group of filamentous fungi, can destroy lung tissue and blood vessels. Regrettably, in the current study, it appears that two out of the three children studied passed away owing to a lack of timely diagnosis and treatment. Kashefi *et al*. reported the successful treatment of PA caused by *A*. *fumigatus* in a child with systemic lupus erythematosus using amphotericin B (50 mg/day) for 19 days [10]. Therefore, an accurate diagnosis of PA infection using paraclinical findings of the patient, such as radiographic images and laboratory results, can play a crucial role, especially in vulnerable children. Timely diagnosis and treatment of PA in children can reduce the risk of complications and mortality rates [14]. The present study has several novel findings, including the identification of three causative agents of PA by *A*. *flavus* in a specialized children’s hospital in Northeast Iran. This study is also the first research investigated in this region during the COVID-19 era. Furthermore, the present study examined the quantitative molecular diagnostic method for detecting *P*. *jirovecii* pneumonia (PJP) in pediatric patients. The present study has some limitations, including a relatively small sample size of children. Furthermore, we lacked comprehensive information about potential underlying diseases and the patients’ medical histories, including previous PA conditions and treatments, and access to serological methods for a more precise diagnosis of this disease.

## Conclusion

Given that PA in pediatric patients has significant potential for morbidity and mortality complications, early diagnosis can be critical in decreasing the fatality rate. Furthermore, antifungal prophylaxis appears to be crucial for improving survival in these patients.

## Data Availability

Written informed consent was obtained from patient’s accompanying individual and are available for provision to the journal on demand.
